# What are the characteristics of participatory surveillance systems for influenza-like-illness?

**DOI:** 10.7189/jogh.13.04130

**Published:** 2023-10-20

**Authors:** Nadege Atkins, Mandara Harikar, Kirsten Duggan, Agnieszka Zawiejska, Vaishali Vardhan, Laura Vokey, Marshall Dozier, Emma F de los Godos, Emilie Mcswiggan, Ruth Mcquillan, Evropi Theodoratou, Ting Shi

**Affiliations:** 1 Center for Population Health Sciences, Usher Institute, University of Edinburgh, Scotland, UK; 2 UNCOVER (Usher Network for COVID-19 Evidence Reviews) Usher Institute, University of Edinburgh, Edinburgh, UK; * Joint first authorship.; † Equal contribution.

## Abstract

**Background:**

Seasonal influenza causes significant morbidity and mortality, with an estimated 9.4 million hospitalisations and 290 000-650 000 respiratory related-deaths globally each year. Influenza can also cause mild illness, which is why not all symptomatic persons might necessarily be tested for influenza. To monitor influenza activity, healthcare facility-based syndromic surveillance for influenza-like illness is often implemented. Participatory surveillance systems for influenza-like illness (ILI) play an important role in influenza surveillance and can complement traditional facility-based surveillance systems to provide real-time estimates of influenza-like illness activity. However, such systems differ in designs between countries and contexts, making it necessary to identify their characteristics to better understand how they fit traditional surveillance systems. Consequently, we aimed to investigate the performance of participatory surveillance systems for ILI worldwide.

**Methods:**

We systematically searched four databases for relevant articles on influenza participatory surveillance systems for ILI. We extracted data from the included, eligible studies and assessed their quality using the Joanna Briggs Critical Appraisal Tools. We then synthesised the findings using narrative synthesis.

**Results:**

We included 39 out of 3797 retrieved articles for analysis. We identified 26 participatory surveillance systems, most of which sought to capture the burden and trends of influenza-like illness and acute respiratory infections among cohorts with risk factors for influenza-like illness. Of all the surveillance system attributes assessed, 52% reported on correlation with other surveillance systems, 27% on representativeness, and 21% on acceptability. Among studies that reported these attributes, all systems were rated highly in terms of simplicity, flexibility, sensitivity, utility, and timeliness. Most systems (87.5%) were also well accepted by users, though participation rates varied widely. However, despite their potential for greater reach and accessibility, most systems (90%) fared poorly in terms of representativeness of the population. Stability was a concern for some systems (60%), as was completeness (50%).

**Conclusions:**

The analysis of participatory surveillance system attributes showed their potential in providing timely and reliable influenza data, especially in combination with traditional hospital- and laboratory led-surveillance systems. Further research is needed to design future systems with greater uptake and utility.

Seasonal influenza causes high rates of illness and mortality around the world. Estimates of yearly respiratory-related deaths range from around 290 000 to nearly 650 000 [1], with over 9.4 million people hospitalised with influenza-related lower respiratory infections in 2017 [2].

Influenza surveillance is defined as “the collection, compilation and analysis of information on influenza activity in a defined population”; its major objective is to lessen the disease's effects by giving public health authorities meaningful data so they may more effectively plan suitable control and intervention measures, allocate resources to healthcare, and provide case management suggestions [3].

Participatory surveillance systems are emerging alongside more traditional forms of disease surveillance. They typically involve people reporting their own health information in real-time using tools such as apps or hotlines [4]. Unlike traditional surveillance systems, which rely on reports from health professionals or laboratory testing based on specific case definitions, participatory surveillance involves individuals sharing information, typically about symptoms rather than diagnoses [4]. As such, they are more sensitive but less specific than traditional surveillance systems and can provide timely information about disease within a population [4,5]. Research has indicated that, in view of influence surveillance, participatory surveillance systems can act as reliable complements to current sentinel surveillance systems [6]. However, these systems have some key challenges, such as representativeness (who chooses to participate), accessibility (availability of internet or smartphone access), and health literacy [4].

Participatory surveillance systems have been recognised as having the potential to play an important role in population-level disease surveillance [4,5]. Research Recommendation 1.1.2 of the World Health Organization (WHO) Public Health Research Agenda for Influenza is to identify the reliability of complementary influenza surveillance systems, such as participatory surveillance, for providing real-time estimates of influenza activity.

There is considerable variation and creativity in participatory surveillance system design worldwide. While research has begun to show that participatory surveillance systems are useful as a complement to other forms of disease surveillance [4,6], there is no synthesis available on this topic. Thus, we aimed to identify the purpose and attributes of participatory surveillance systems for influenza-like illness (ILI) to provide information to decision-makers and organisations (such as WHO) interested in implementing, establishing or enhancing their own participatory surveillance systems.

## METHODS

Prior to conducting this review, we developed a study protocol based on the Preferred Reporting Items for Systematic Reviews and Meta-Analysis for Protocols 2015 (PRISMA-P 2015) guidelines (Appendix 1 in the **Online Supplementary Document**) and later followed the PRISMA 2020 guidelines in conducting the study.

We systematically searched EMBASE, Global Health, MEDLINE, and medRxiv on 14 December 2022 to identify relevant studies evaluating influenza and ILI participatory surveillance systems. We previously created comprehensive search strategies for each database using keywords and alternative terms derived from literature scoping searches (eg, “influenza” and “surveillance” (Appendix 2 in the **Online Supplementary Document**). We imported the search results from Embase, Global Health, and MEDLINE into Covidence (Covidence, Melbourne, Australia) and those from medRxiv into Mendeley, version 1.19.4. (Elsevier, Amsterdam, Netherlands), as Covidence does not support medRxiv citations. We then conducted automatic deduplication, followed by manual removal of any remaining duplicates any duplicates missed by the software.

Two reviewers then independently screened the titles and abstracts of retrieved article followed by full-texts of potentially eligible studies, according to pre-developed eligibility criteria (**Table 1**). Conflicts were resolved by a third independent reviewer. A list of the excluded studies and the reasons for their exclusion during the full-text reading stage is provided in Appendix 3 in the **Online Supplementary Document**.

**Table 1:** Eligibility criteria used for the review

Inclusion criteria	Exclusion criteria
Studies reporting on influenza/ILI participatory surveillance systems	Studies reporting on non- participatory surveillance systems
Studies with information on any of the following characteristics (objectives, attributes, ethics) of influenza/ILI participatory surveillance systems	Studies with no/inadequate information on the characteristics of influenza/ILI participatory surveillance systems
Primary studies (cohort, cross-sectional)	Secondary studies (they were searched for additional eligible references)

ILI – influenza-like illness

Two reviewers then independently extracted data from each eligible study using a standard data extraction form, previously developed based on the Cochrane guidelines [7]. In an iterative process, the form was first piloted on three studies and adapted to ensure that all relevant data were extracted. We extracted data regarding category (studies reporting results from participatory surveillance systems or studies evaluating participatory surveillance systems), study location, study aim, definition of influenza definition used, and objectives and attributes of the participatory surveillance system.

We performed quality assessment of the included studies with the Joanna Briggs Institute (JBI) Critical Appraisal tool [8], composed of questions related to selection of study participants, measurement of exposures and outcomes, and adjustment for potential confounders. Each question was answered with either “Yes”, “No”, “Unclear” or “Not applicable”. We calculated the percentage of “Yes” responses among all questions to attain comparable quality scores among the selected studies, so the overall quality score for each study ranged from 0 to 100 (80-100 = high quality, 50-80 = moderate quality, <50 = low quality). Due to time constraints, each study’s quality was assessed by a single reviewer.

We conducted a narrative synthesis following guidance from Popay et al. [9] and the Centre for Reviews and Dissemination [10].

## RESULTS

**Figure 1:**
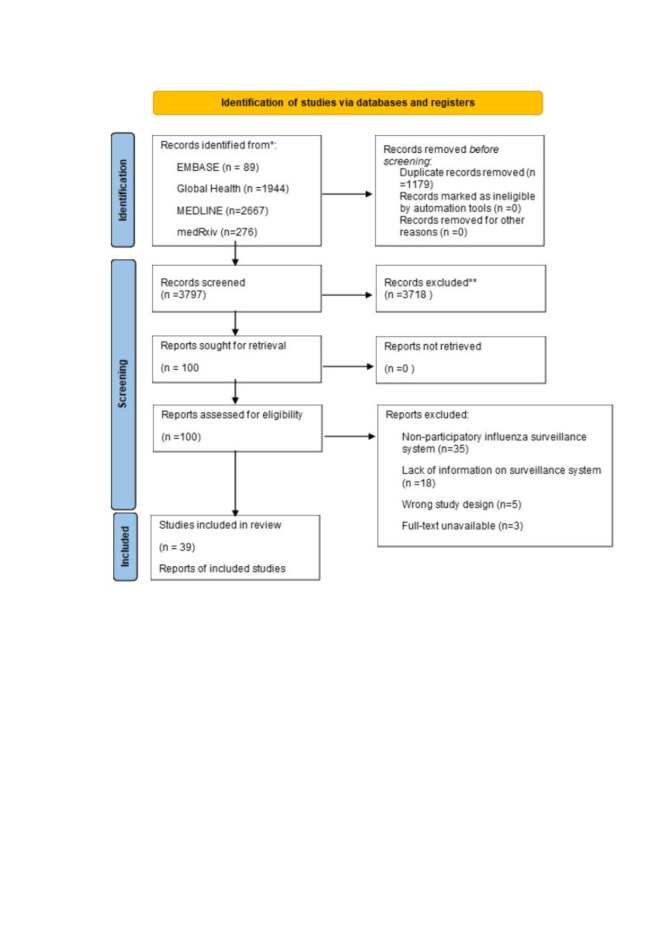
PRISMA flow diagram of screening process.

The search retrieved 4976 studies, 1179 of which were discarded as duplicates. Two reviewers independently screened the titles and abstracts of the remaining 3797 studies, with a third reviewer resolving conflicts. After excluding 3718 studies, two independent reviewers read the full texts of the remaining 100 articles, 61 of which were excluded as they covered non-participatory surveillance systems (n = 35), lacked information on features of surveillances systems (n = 18), had a wrong study design (n = 5), or did not have a full-text available (n = 3). We finally included 39 studies meeting the eligibility criteria (**Figure 1**). These studies encompassed 18 geographical locations; eighteen were conducted in the European Region, twelve in the Region of Americas, and nine in the Western Pacific Region (Table S1 in the **Online Supplementary Document**). We included three cohort studies, which were rated as high quality, with average score of 85%, and 36 cross-sectional/observational case studies, which were all of moderate quality, with an average score of 72% (Tables S2-S3 in the **Online Supplementary Document**).

### ILI participatory surveillance systems and their objectives

The objectives of 18 of the 26 participatory surveillance systems we identified were explicitly stated by the authors (**Table 2**).

**Table 2:** ILI participatory surveillance systems and objectives

Author (year)	Location	Name of System	Objective(s)
Baltrusaitis et al. (2017) [11], Baltrusaitis et al. (2019) [12], Chunara et al. (2015) [13]	USA	FNY	To assess influenza attack rates in specific cohorts.
Bexelius et al. (2010) [40]	Sweden	IVR System with web-based questionnaires for population-based infectious disease reporting	To collect outbreak data in a timely fashion.
Carlson et al. (2013) [37], Dalton et al. (2009) [39], Dalton et al. (2011) [44], Dalton et al. (2015) [34]	Australia	FluTracking	
Debin et al. (2013) [45]; Guerrisi et al. (2018) [14]	France	GN	To estimate ILI frequency among the GN cohort in France and identify the factors associated with ILI infections to provide aid to epidemiological and public health monitoring and research.
Desroches et al. (2021) [15], Lee et al. (2021)[16]	Canada	Fluwatchers	To track community ILI activity and to capture the spread of ILI among individuals who do not seek medical care.
Elliot et al. (2009) [32]	UK	National Pandemic Flu Service	To compare a self-sampling approach to sentinel surveillance.
Fujibayashi et al. (2018) [17]	Japan	Flu-Report	To monitor influenza activity more easily and faster than the conventional surveillance systems.
Kamimoto et al. (2013) [18]	USA	Telephone surveys using questionnaire	To estimate overall population burden of influenza by measuring incidence of ILI, influenza diagnosis by healthcare providers, use of rapid testing and treatment of influenza with antiviral medicines.
Kjelso et al. (2015) [35]	Denmark	Influmeter	To monitor self-reported ILI in the general population.
Kim et al. (2019) [19]	South Korea	Fever Coach	To provide actionable, tailored information for fever management in children younger than five years.
Loubet et al. (2016) [31]	France	GGNET	to describe the epidemiology of seasonal influenza during influenza season among pregnant women
Lucero et al. (2017) [20]	USA	VA TT ESSENCE	To track influenza activity in VA during H1N1 influenza pandemic. To evaluate ILI trends in combined VA and US Department of Defense patient populations.
Lwin et al. (2020) [21]	Singapore	FluMob	To serve as a complementary surveillance system for influenza in addition to existing surveillance systems designed exclusively for healthcare workers.
Marmara et al. (2015) [33]	Malta	Survey 1 and Survey 2	To investigate under-reporting of ILI compared to GP reports.
Perrotta et al. (2017) [47]	Italy	Influweb	To provide epidemiological information directly from the general population using self-reports from volunteers completing weekly surveys over the flu season.
Paixao et al. (2017) [29]	Portugal	Surveillance based/nested within ENVIRH	To support existing surveillance system/test additional surveillance. To test a surveillance system for respiratory infections in children. To check if parents’ collaboration, coupled with a sample collection by a dedicated team, could add important information to that obtained from the National Programme for Flu Surveillance.
Vandensjick et al. (2013) [22], de Lange et al. (2013) [46]	Belgium, The Netherlands	GIS	To monitor ILI in the general population via the internet.

FNY – Flu Near You, IVR – Interactive voice response, GN – Grippe Net, GGNET – Grossesse-GrippeNet (Pregnancy-GrippeNet), ILI – influenza-like illness, VA TT – Veterans Affair Telephone Triage, ENVIRH – Environment and Health in children day care centers, GIS – Great Influenza System, GP – general practitioner

### Attributes of participatory surveillance systems

The most frequently reported system property was the correlation/concurrence with other surveillance systems. Almost all studies reported a statistically significant, moderate, or strong correlation between ILI incidences captured by the participatory systems and trends recorded by the comparators [11-27]. Only Prieto et al. [28] found a negative correlation (r = -0.3) between the number of ILI cases reported to their Mi Gripe system and the number of reported, laboratory-confirmed cases of influenza published by the Pan American Health Organization.

Fifteen studies analysed the utility of the participatory system and reported multiple uses, apart from collecting epidemiological data on the ILI incidence. The systems allowed access to specific populations, such as children [19,29,30], pregnant women [31], and healthcare workers [21], and access to information on healthcare-seeking behaviour [16,32,33]. Participatory systems like FluWatchers or FluTracking were used to collect information about vaccination uptake and effectiveness [16,34], while the School Health Surveillance System (SHSS) system was designed to provide data on school absenteeism [30].

Eleven studies explored timeliness. Most reported prompt detection of changes in baseline ILI activities by participatory systems, ahead of the pattern changes detected by the reference systems [16,20,27]. Most participatory systems’ users disclosed information about their health status within three days from the weekly reminders being distributed [17,28,35,36] or within three days from the symptoms’ onset [17].

Ten studies reported on the systems’ representativeness. Several studies reported the bias towards female users [14-16,23,35,37] and better educated populations. One study reported bias towards younger users [23], while two studies noted that users were older compared to the general population [14,38]. Despite these inconsistencies, the extremities of age groups were the most underrepresented, except in the case of Kim et al., whose study was designed to target the paediatric population [16,19,22,35].

Eight studies provided data on participatory systems’ acceptability. Except for the study by Vandendijck et al. on a Belgian cohort of the Great Influenza System (GIS) users [22], all studies consistently reported high acceptability expressed as high response (74%-78%) and retention rates (79%-80%) for FluWatchers. Australian studies on FluTracking also noted increasing number of participants in the subsequent rounds of recruitment [37,39].

Only a few studies analysed systems’ flexibility, reporting the ability to either expand a system’s design to capture more data over the study period [20,27] or perform adjustments in response to the users’ changing needs [28,40].

There were few data on the systems’ sensitivity; a few studies reported that participatory systems were able to capture epidemic peaks up to three weeks before they were detected by the reference surveillance systems [19,24,35,38,41].

A few studies discussed the systems’ simplicity, mostly reporting various ways of facilitating participants’ contribution, like the availability of a dedicated, free-of-charge phone line for parents [29], an easily accessible web-based portal, or applications compatible with operating systems commonly used on mobile devices [21]. The studies highlighted that intuitive software and automated maintenance of the system improved researchers’ experience with the National Health Service (NHS) Direct and Mi Gripe, respectively [28,42].

Six studies analysed system stability, reporting on disruptions in the systems’ operation taking up to two months for the FluMob, which resulted in data loss during the 2007 influenza outbreak [21]. Meanwhile, the stable Japanese SHSS benefited from the established system of school absence reporting, while NHS Direct was protected from data loss by regular service of the software and data transmission modalities [30,42].

Only two studies monitored completeness of the data, returning inconsistent observations. While more than half of Mi Gripe application did not send reports, data circulated within the NHS Direct surveillance system were complete [28,42].

### Approaches to collecting and storing data

Web-based data collection systems included online questionnaires, study-specific websites, dedicated mobile applications, and email prompts with links to the survey. Other conventional and time-tested technologies, such as telephonic interviews, interactive voice response systems, and text messages were also used. While most systems relied on the self-reporting of symptoms, smart thermometers directly sent temperature readings via an accompanying mobile application.

Only two studies [19,21] reported on data storage. Fever coach data was stored in real time databases that were updated daily, while FluMob data was also stored similarly in an accessible central database updated in real time (Table S5 in the **Online Supplementary Document**).

### Study approval

Less than one third of the studies evaluating participatory surveillance systems confirmed seeking participants’ consent for their participation in the study (n/N = 11/39 (28.2%)), and less than half (n/N = 15/39 (38.46%)) reported seeking prior approval by an Ethics Board (Table S6 in the **Online Supplementary Document**).

### Recruitment and retention of participants

Of 22 systems that reported on recruitment of participants, only three (13.63%) reported using a sampling method for data collection. Most other systems randomly recruited participants via email, text messages, push-notifications on mobile phones, and social media platforms. Press releases, television advertisements, website notifications, and posters were also used to publicise the system and invite participants. Only a few systems (n/N = 8/26 (30.76%)) reported efforts taken to retain participants in the system by sending weekly reminders through SMS, app notifications, or email newsletters (**Table 3**).

**Table 3:** ILI surveillance systems’ recruitment and retention of participants strategies

Author (year)	Location	System	Method used for recruitment of participants	Method used for retainment of participants
Baltrusaitis et al. (2017) [11], Baltrusaitis et al. (2019) [12], Chunara et al. (2015) [13]	USA	FNY	Participants are sent a short weekly survey via email or a smartphone push notification asking if they experienced any of 10 select symptoms.	N/A
Bexelius et al. (2017) [40]	Sweden	IVR System with web-based questionnaires for population-based infectious disease reporting	Random invitation to 14 000 inhabitants.	N/A
Biggerstaff et al. (2012) [43]	USA	BRFSS	Participants were recruited from the general population by random-digit dialing.	N/A
Carlson et al. 2013 [37], Dalton et al. (2009) [39], Dalton et al. (2011) [44]	Australia	Flu tracking	Use of social media tools (eg, Facebook and Twitter), media releases.	N/A
Dalton et al. (2015) [34]	Australia	Flu tracking	Facebook advertising; media coverage. Recruitment through previous participants.	Cohort maintained and boosted every year by annual recruitment drive from March to May. The most successful recruitment strategy in 2013 and 2014 was recruitment through previous participants.
Debin et al. (2013) [45], Guerrisi et al. (2018) [14]	France	GN	Press releases.	N/A
de Lange et al. (2015) [46]	The Netherlands	GIS	Yearly press releases inviting to participate addressed to Dutch and Belgian populations.	Weekly e-mail reminders with links to the surveys.
Fujibayashi et al. (2018) [17]	Japan	Flu-Report	Volunteers were recruited via TV advertisements, articles on the internet, and posters at cooperating medical facilities.	Monthly reminders were sent via a message on the iPhone screen to remind participants to report any influenza infection.
Kim et al. (2019) [19]	South Korea	Fever Coach	The researchers promoted the app by: providing free coffee coupons for randomly selected users who downloaded the app and shared their experience and; providing free online health consultation. After reaching 100 000 downloads in May 2016, there have not been active marketing activities, but parents who used the app have voluntarily spread the word on the internet and in their local communities.	N/A
Kjelso et al. (2015) [35]	Denmark	Influmeter	The system was promoted through several forms of Danish news media and through the Influmeter website. Participants could enrol through the website and report their own symptoms together with symptoms on behalf of friends or relatives, including children in their household.	N/A
Lee et al. (2014)[38]	Tuen Mun District, Hong Kong	E-community Surveillance System for Influenza-Like Illness	Postal invitations were sent for online registration on a study website, E-community Surveillance System for Influenza- Like Illness	A weekly reminder was emailed, and an incentive (coffee coupon) was offered for every 10 weekly updates
Loubet et al. (2016) [31]	France	GGNET	Articles were published on websites. Two clinical research networks and the involved maternity hospitals displayed advertising posters in their waiting rooms to recruit pregnant women.	N/A
Lwin et al. (2020) [21]	Singapore	Flu Mob	Convenience sampling was used to recruit clinical and non-clinical hospital staff from all departments via mass emails.	Participants were sent app notifications on a weekly basis and prompted to complete a questionnaire on acute respiratory symptoms through the FluMob app.
Marmara et al. (2015) [33]	Malta	Survey 1 and Survey 2	Individuals were invited to participate in the study through a telephone survey.	N/A
Paixao et al. (2014) [29]	Portugal	Surveillance project based/ nested within ENVIRH	Recruitment via Day care centres.	N/A
Perrotta et al. (2017) [47]	Italy	Influweb	The yearly study is disseminated among the general population at the beginning of each flu season through a number of press releases.	N/A
Prieto et al. (2017) [28]	Guatemala	Mi Gripe	The heads of 189 households were recruited during randomly selected home visits. Participants used text messages or an app to report symptoms of ILI at home, the ages of the ILI cases, if medical attention was sought, and if medicines were bought in pharmacies.	Weekly reminders were sent to participants and those who sent reports were compensated with phone credit.
Rehn et al. (2014) [48]	Sweden	IMS	Recruitment via press releases /resulting media attention plus interpersonal communication through social media channels.	N/A
Stockwell et al. (2014) [36]	NYC, USA	MoSAIC	Households were identified by contacting a random sample of participants who had taken part in a large population-based survey of an urban, primarily immigrant Latino community.	Households were visited monthly to promote retention. As an incentive to participate, household reporters received US$20 each month if they responded to at least 75% of the month’s text messages.
Takahashi et al. (2001) [30]	Japan	Japanese School Health Surveillance System for Influenza	All school children included automatically.	N/A
Tilston et al. (2010) [26]	Great Britain (England, Wales, Scotland and Northern Ireland)	UK Flu Survey	With the help of a publicity campaign involving television, radio, and newspaper coverage and word of mouth, participants were recruited. Registration for the UK flusurvey took place through the web page http://www.flusurvey.org.uk	An email newsletter was sent to participants each week to remind them to complete the symptoms questionnaire.
Vandensjick et al. (2013) [22]	Belgium	GIS	Participation was carried out via registration on the website www.degrotegriepmeting.nl. People who registered were invited by weekly emails to participate in an online symptom questionnaire.	N/A
van Noort et al. (2015) [27]	The Netherlands, Belgium, Portugal, and Italy	Influenzanet	Participants were recruited from the general population by completing an intake questionnaire on one of the national websites.	N/A

FNY – Flu Near You, IVR – Interactive Voice Response, BRFSS – Behavioral Risk Factor Surveillance System, GN – Grippe Net, GIS – Great influenza Survey, GGNET – Grossesse-GrippeNet (Pregnancy-GrippeNet), ENVIRNH – Environment and Health in children day care centers, IMS – Internet-based monitoring system, MoSAIC – Mobile Surveillance for Acute Respiratory Infections and Influenza-Like Illness in the Community, ILI – influenza-like illness

### Adjustment for potential bias and confounders

Fifteen studies reported on adjusting for the potential bias, while only five adjusted for confounders mostly sex, age, or localisation [14,25,33,40,45] (Table S7 in the **Online Supplementary Document**).

## DISCUSSION

Through this rapid review, we summarised the characteristics of participatory surveillance systems for influenza worldwide based on 39 eligible studies from 18 countries on 26 participatory surveillance systems. Twenty-one (80%) of the included studies reported on participatory surveillance systems’ correlation with other surveillance systems, 10 (38%) on acceptability, 16 (62%) on representativeness, 15 (58%) on timeliness and utility, five (19%) on flexibility, and four (15%) simplicity and stability. There was limited data available on the completeness of the systems with only two (8%) studies reporting on this attribute.

Participatory influenza surveillance systems were often found to be comparable [32,40] or, in a few instances, superior to existing sentinel surveillance systems [29,35] in providing advanced warning of seasonal influenza activity. However, while these systems were reported to have high sensitivity for influenza detection, their specificity may be variable depending on the syndromic definition of ILI used.

The systems’ ability to adapt to changing user demographics, data requirements, and improved user experience suggests the ability of participatory surveillance systems in adjusting to the changing demands of a public health threat. However, only a few reported on this attribute, necessitating more research.

Most systems used a web-based portal for data collection (n/N = 14/26), five used telephone surveys, three used mobile apps, and two reported mixed approaches comprising mobile and web-based platforms or telephone surveys and laboratory sample data. The data storage facilities were recorded for only two systems which used mobile apps for data collection – Flu mob [21] and Fever coach [19]. Most systems did not attain ethical approval from the regional Ethical committee for data collection. Of 22 systems that reported on recruitment of participants, only three reported using a sampling method for data collection, while the remaining ones mostly randomly recruited participants by sending invitations via telephone, text messages, or web-based platforms. Recruitment was bolstered by posters, website notifications, and television advertisements. Less than a third of systems reported efforts taken to retain participants via weekly reminders sent through SMS, app notifications, or email newsletters. Data on adjustment for bias were reported for 13 systems and data on adjustment for confounders for four systems.

This rapid review confirms the sustainability of the systems that were set up almost two decades ago and have been operating for more than a decade, like the GIS [22,46]. Thus, it adds to the existing body of evidence that participatory systems evolved into a stable form of surveillance on the national, regional and global levels [6]. Next, participatory systems have become a valid source of data contributing to modelling and simulating surveillance systems in a wider interdisciplinary context, involving a broader range of stakeholders. Importantly, studies on the Australian FluTracking system also evaluated its operation, which has been identified by the recent review as an important aspect for informing modern participatory systems [16].

Fifteen out of 39 studies selected for the review reported seeking ethical clearance for the systems, while even a smaller proportion (n = 11) reported obtaining subjects’ approval to participate in the study. These weaknesses are also discussed by a review or research ethics within the Influenzanet consortium, which confirmed that most of its member countries sought for the approvals from the Research Ethics Committees and protected personal data [49]. However, the low proportion of identified studies satisfying ethical requirements and subjects’ rights may reflect the complexity and differentiated awareness of biomedical ethics regarding large-scale public health interventions in the digital era. These concerns are also comprehensively addressed in recent literature, which offers an ethical framework developed specifically for participatory surveillance systems designed for human health [49].

We also identified studies re-using routinely available medical data collected alongside triaging the patients within the emergency or out-patient facilities in attempts to use this information for influenza surveillance [41,42,50]. This approach explores options to double-use information collected within healthcare systems both for medical purposes and to support and enhance surveillance. Although capable of addressing surveillance aspects of healthcare systems and reported as non-traditional surveillance systems elsewhere [6], this approach should be taken with caution and carefully examined to ensure that aims specific for public health are also satisfied.

### Strengths and limitations

The strengths of this review are its robust systematic review methodology, development of a detailed protocol to ensure transparency, creation of comprehensive search strategies, and the use of a wide range of databases. We also included unpublished literature to reduce the impact of publication bias. Another strength is the broad eligibility criteria — rather than restricting the search to studies that explicitly claimed to be “participatory”, we screened all reports on influenza surveillance systems and selected those that were participatory. This helped include systems like the Behavioral Risk Factor Surveillance System (BRFSS) or NHS Direct that are also used for non-ILI-related surveillance.

Among the included studies, we found systems to vary widely in their objectives, attributes, and harboured technology, making their evaluation in this review useful for a wide range of surveillance scenarios. We also looked at the lesser-known aspects of these systems,such as whether they acquired ethical approval and how they collected and stored data, which can be useful in the development of future systems.

However, this study also has some limitations. First, we could only conduct a narrative syntheses, which are inherently prone to bias based on reviewers’ interpretations [51]. Second, the assessment of study quality was subjective and challenging due to considerable heterogeneity among the included studies. Further, owing to time constraints, the quality assessment was only performed by a single reviewer. The choice of appropriate quality appraisal tool initially proved to be challenging, considering the diversity of study designs of included studies and their research questions, and the fact that most studies did not explicitly state their study design. We thus assessed those reported as cohort studies using the JBI tool for cohort studies and the remaining ones using the JBI tool for cross-sectional studies. Given that most studies examining participatory surveillance systems did not use a valid method to identify the condition (such as laboratory-confirmed diagnosis of influenza) or measure the condition reliably, they were rated to be moderate in quality. Nevertheless, this is a limitation of participatory surveillance systems in general, and save for the above two characteristics, most studies were otherwise scientifically robust.

We only included studies published in English, possibly introducing language bias. Finally, we excluded studies that focused exclusively on the use of participatory surveillance systems for vaccine monitoring and not for tracking ILI or severe acute respiratory infections (SARI). Further studies on the use of participatory surveillance systems in assessing vaccine uptake and effectiveness will be useful in discovering wider applications of these systems.

Our findings can support further research in participatory surveillance systems and provide information for public health policy makers looking to establish additional surveillance. Participatory surveillance can be a useful complement to existing sentinel surveillance systems. However, future research could gather data from more representative samples of the population to establish acceptability of any participatory system, especially due to their ability to reach populations which are less likely to be included in traditional surveillance. Here we demonstrated the need for acceptability, correlation, and timeliness of participatory surveillance, and the potential effects of unstable systems. As a cost-benefit analysis would also be of interest to policy makers, future analyses should consider the aspects of cost and simplicity of running and maintaining any system.

## CONCLUSIONS

Participatory surveillance systems possess considerable potential in providing timely and reliable influenza surveillance data. Certain limitations, such as poor representativeness of the user population, unavailability of complete data, disparate access to the surveillance system, and inadequate ethical clearance may prevent participatory systems from substituting physician-, hospital-, and laboratory-led surveillance systems. Nevertheless, they may be valuable in complementing traditional surveillance systems, especially for vulnerable populations who may not seek timely care, for remote and underserved regions, or for periods when traditional systems are overburdened or lacking. Moreover, participatory surveillance systems can aid in understanding healthcare seeking behaviour.

While we highlighted the characteristics of existing participatory surveillance systems in this review, further research on what makes participatory surveillance systems successful will help design future systems with greater uptake and utility for influenza surveillance. Given that influenza threatens global public health security, timely and accurate surveillance is of critical importance to protect those most at risk.

## Additional material


Online Supplementary Document
