# Supplementary material for: What are the characteristics of participatory surveillance systems for influenza-like-illness?
Source: J Glob Health. 2023 Oct 20;13:04130. doi: 10.7189/jogh.13.04130 (PMC10587643; doi:10.7189/jogh.13.04130)
Supplement: Online Supplementary Document [file jogh-13-04130-s001.pdf]

Appendix S1: Study Protocol

Research Question: What are the characteristics of the current participatory surveillance systems for influenza and influenza-like-illnesses?

Introduction

The World Health Organization (WHO) (2022) defines participatory surveillance as 'the bi-directional process of receiving and transmitting of health-related data through direct engagement of the general population'. These surveillance studies use various data-collection tools such as emails, mobile applications, SMS, and hotlines to collect data. Participatory surveillance systems play a vital function in allowing for the early detection, monitoring and prevention of public health threats, such as that of influenza virus.

In the past decade, influenza participatory surveillance systems such as the web-based surveys *FluTracking* and *InfluenzaNet*, have emerged. These surveillance systems have been proven to be accurate and reliable as their relative intensities of influenza epidemics have been consistent with that reported by sentinel doctors (WHO,2022). In addition, influenza participatory surveillance systems provide near real-time information on the burden and severity of influenza especially in symptomatic individuals who may not seek healthcare for their illnesses, thus filling a data gap and complementing data gathered from traditional surveillance systems in countries where these systems have been implemented.

Rationale

The WHO public health research agenda for influenza has recommended that research be conducted on the characteristics of current participatory surveillance systems for influenza and influenza-like-illnesses. It is imperative that such research be conducted as it will provide vital information to public health institutions interested in implementing participatory surveillance systems for influenza and influenza-like-illnesses. This research will also highlight objectives and attributes of effective influenza participatory surveillance systems.

Objectives

This review aims to systematically identify, synthesize, and critically analyse existing evidence on current influenza and influenza-like-illnesses participatory surveillance systems across the world.

The objectives of the review are to :

- To summarize the objectives and attributes (acceptability, representativeness, timeliness, correlation with traditional surveillance systems, data quality and completeness, flexibility, sustainability, utility) of current influenza and influenza-like-illnesses participatory surveillance systems.
- To synthesise their approaches to collecting and storing data.
- To investigate if they have sought approval from Ethics Boards or offer Informed Consent.
- To characterize the recruitment and retainment of participants.
- To review if they assess or adjust for potential bias and confounders.

Method
The proposed research will take the format of a rapid review.

Eligibility criteria

**Inclusion criteria**	**Exclusion criteria**
Studies reporting on influenza/ influenza-like-illnesses participatory surveillance systems	Studies reporting on surveillance systems that are not influenza/ influenza-like-illnesses participatory surveillance systems
Studies reporting on human influenza/influenza-like-illnesses participatory surveillance systems	Studies reporting on plant or animal surveillance
Studies with adequate information on the characteristics (objectives, attributes, ethics) of the influenza/influenza-like-illnesses participatory surveillance systems	Studies with inadequate information on the characteristics of the influenza/influenza-like-illnesses participatory surveillance systems

Information Sources
In the month of December, MEDLINE, EMBASE, and medRxiv electronic databases will be searched to identify relevant articles written in English. Both published and unpublished literature will be retrieved so as to avoid publication bias. Reference lists of the included literature will also be searched for identification of potentially relevant papers.

Search Strategy
The search strategy will include key and alternative terms related to the research topic which will be derived from literature scoping searches. These search terms will include terms related to participatory surveillance and influenza. Additionally, a senior librarian with experience in supporting systematic reviews will be consulted to ensure that a comprehensive search strategy is developed. The results of each search will be clearly documented in the Appendix.

Study records:

Data Management
Records will be managed using COVIDENCE; a specific software used for management of systematic reviews. Duplicate records will also be removed using the software.

Selection process
Two independent reviewers will perform title abstract screening and full-text screening of the records retrieved, and those meeting the study's eligibility criteria will be retained. A PRISMA (Preferred Reporting Items for Systematic Reviews and Meta Analysis) flowchart will be provided showing the study selection process.

Data collection process
For studies that fulfill the inclusion criteria, two independent reviewer will extract data using a tailored standard data extraction form.

Data items
Data to be extracted will include information related to the study location, objectives and attributes of the surveillance system.

Data synthesis:
Data will be synthesized using narrative synthesis.

References
World Health Organization (2022) *Invitation for public comment: Draft Best Practices for the Design, Implementation, Analysis and Reporting of Participatory Surveillance for Influenza-like Illness*. Available at :

https://www.citethemrightonline.com/sourcetype?docid=b-9781350927964&tocid=b-9781350927964-34

Appendix S2: Search Strategies

Ovid MEDLINE(R) and In-Process, In-Data-Review & Other Non-Indexed Citations <1946 to December 13, 2022>

1	Influenza, Human/	56387
2	((influenza* or flu or influenza) adj2 illness).ti,ab,tw.	4795
3	1 or 2	57933
4	exp Population Surveillance/	74237
5	(evaluat* or perform* or effect*).ti,ab,tw.	12523072
6	4 and 5	26718
7	(surveillance and (evaluat* or perform* or effect*)).ti,ab,tw.	102981
8	6 or 7	117435
9	3 and 8	2671
10	exp animals/ not humans.sh.	5068872
11	9 not 10	2667

Embase <1980 to 2022 Week 49>

1	Influenza, Human/	26,766
2	((influenza* or flu or influenza) adj2 illness).ti,ab,tw.	6,102
3	1 or 2	31,944
4	exp Population Surveillance/	145
5	(evaluat* or perform* or effect*).ti,ab,tw.	15,805,290
6	4 and 5	62
7	(surveillance and (evaluat* or perform* or effect*)).ti,ab,tw.	155,373
8	6 or 7	155,399
9	3 and 8	1,527
10	exp animals/ not humans.sh.	28,567,109
11	9 not 10	89

Global Health <1973 to 2022 Week 49>

1	influenza/	23159
2	((influenza* or flu or influenza) adj2 illness).ti,ab,tw.	3716
3	1 or 2	24817
4	exp surveillance/	56368
5	(evaluat* or perform* or effect*).ti,ab,tw.	2103864
6	4 and 5	28585
7	(surveillance and (evaluat* or perform* or effect*)).ti,ab,tw.	48893
8	6 or 7	65330
9	3 and 8	2011
10	Animals/ not (Animals/ and Humans/)	691364
11	9 not 10	1944

medRxiv

Topic 1: influenza

Influenza-like illness
Severe acute respiratory infections
Topic 2 :Surveillance
Monitoring
Participatory
Results:106

Appendix S3: Excluded studies

Database ID/ doi	Author (year)	Title	Reason for Exclusion
PMID: 28144681	Adnan et al. 2017	Electronic Sentinel Surveillance of Influenza-like Illness. Experience from a pilot study in New Zealand	Non-participatory surveillance systems
PMID: 25189347	Al Tawfique et al 2014	Surveillance for emerging respiratory viruses	Wrong study design
PMID: 25037278	Araz et al. 2014	Using Google Flu Trends data in forecasting influenza-like-illness related ED visits in Omaha, Nebraska	Non-participatory surveillance systems
PMID: 51621627	ArranzIzquierdo et al. 2012	Syndromic surveillance of Influenza-like illness in primary care: A complement to the sentinel surveillance network for periods of increased incidence of Influenza	Non-participatory surveillance systems
PMID: 218260	Assaad et al. 1978	[International influenza surveillance (author's transl)]	Full text unavailable
PMID: 34225760	Baaees et al. 2021	Community-based surveillance in internally displaced people's camps and urban settings during a complex emergency in Yemen in 2020	Non-participatory surveillance systems
PMID: 31823763	Babakazo et al. 2019	Evaluation of the influenza sentinel surveillance system in the Democratic Republic of Congo, 2012-2015	Non-participatory surveillance systems
PMID: 24613818	Bajardi et al. 2014	Determinants of follow-up participation in the Internet-based European influenza surveillance platform Influenzanet	No information on system features
N/A	Baltrusaitis et al. (2021)	Missing data matters in participatory syndromic surveillance systems: comparative evaluation of missing data methods when estimating disease	Lack of information on system features
PMID: 20093314352	Baker et al. 2009	Special Issue: Pandemic H1N1 influenza lessons from the southern hemisphere	Not a research paper
PMID: 21135655	Barr et al. 2011	A process evaluation of an active surveillance system for hospitalized 2009-2010 H1N1 influenza cases	Non-participatory surveillance systems

PMID: 27117107	Bednarska et al. 2016	[Influenza surveillance]	Wrong language
PMID: 25822719	Budgell et al. 2015	Evaluation of two influenza surveillance systems in South Africa	Non-participatory surveillance systems
PMID: 19845471	Carneiro et al 2009	Google trends: a web-based tool for real-time surveillance of disease outbreaks.	Lack of information on system features
PMID: 21471947	CDC 2011	Assessment of ESSENCE performance for influenza-like illness surveillance after an influenza outbreak--U.S. Air Force Academy, Colorado, 2009	Non-participatory surveillance systems
PMID: 12166298	Cooper et al. 2008	Use of NHS Direct calls for surveillance of influenza--a second year's experience	Full text unavailable
PMID: 25391010	Czarkowski et al. 2014	Influenza in Poland in 2011-2012 and in 2011/2012 and 2012/2013 epidemic seasons	Non-participatory surveillance systems
PMID: 35681199	deFougerolles et al. 2022	National influenza surveillance systems in five European countries: a qualitative comparative framework based on WHO guidance	Lack of information on system features
PMID: 22230244	Dugas et al. 2012	Google Flu Trends: correlation with emergency department influenza rates and crowding metrics.	Lack of information on system features
PID:25788252	Eliott et al. (2015)	Self-sampling for community respiratory illness: a new tool for national virological surveillance	Lack of information on system features
PMID: 14728165	Espino et al. 2003	Telephone triage: a timely data source for surveillance of influenza-like diseases.	Non-participatory influenza surveillance system
PMID: 20163337251	Feldblyum & Segal, 2015	Seasonal and pandemic influenza surveillance and disease severity	Lack of information on system features
PMID: 20063054256	Formenty et al. 2006	Emergent pathogens, international surveillance and international health regulations (2005)	Lack of information on system features
PMID: 372069621	Gesualdo et al. 2013	Influenza-like illness surveillance on twitter through automated learning of naive language	Non-participatory surveillance systems

PMID: 18172149	Ghosh et al. 2008	Active influenza surveillance at the local level: a model for local health agencies	Non-participatory surveillance systems
PMID: 34563151	Guerche-Seblain et al. 2021	Comparison of influenza surveillance systems in Australia, China, Malaysia and expert recommendations for influenza control	Lack of information on system features
PMID: 31272411	Guerrisi et al 2019	Factors associated with influenza-like-illness: a crowdsourced cohort study from 2012/13 to 2017/18	Lack of information on system features
PID:36073312	Hammond et al. (2022)	Influenza surveillance systems using traditional and alternative sources of data: A scoping review	Wrong study design
PMID: 1056966	Hart et al. 1975	A programme for collaborative influenza surveillance. A report of a working group of the Public Health Laboratory Service	Non-participatory surveillance systems
PMID: 24717637	Hayward 2014	Comparative community burden and severity of seasonal and pandemic influenza: results of the Flu Watch cohort study.	Lack of information on system features
PMID: 21586265	Hulth & Rydevik, 2011	Web query-based surveillance in Sweden during the influenza A(H1N1)2009 pandemic, April 2009 to February 2010	Non-participatory surveillance systems
PMID: 21500061	Jaeger et al. (2011)	GotFlu channel: an online syndromic surveillance tool supporting college health practice and public health work	Lack of information on system features
PMID: 30958817	Kalimeri 2019	Unsupervised extraction of epidemic syndromes from participatory influenza surveillance self-reported symptoms	Lack of information on system features
PMID: 27772674	Kannan et al. 2016	Geolocalization of Influenza Outbreak Within an Acute Care Population: A Layered-Surveillance Approach.	Non-participatory surveillance systems
https://doi.org/10.1111/j.1467-985X.2012.01025.x	Kavanagh 2012	Syndromic surveillance of influenza-like illness in Scotland during the influenza A H1N1v pandemic and beyond.	Non-participatory influenza surveillance system
PMID: 31705633	Kishore et al. 2020	Flying, phones and flu: Anonymized call records suggest that Keflavik International Airport introduced pandemic H1N1 into Iceland in 2009	Non-participatory surveillance systems
PMID: 28928112	Koppeschaar et al (2017)	Influenzanet: Citizens Among 10 Countries Collaborating to Monitor Influenza in Europe	Lack of information on system

			features
PMID: 20853089	Korte et al. 2010	[Validation of a syndromic surveillance system of acute respiratory tract diseases in preschools of Schleswig-Holstein (SHARE)]	Lack of information on system features
PMID: 20735942	Lajous et al. 2010	Mobile messaging as surveillance tool during pandemic (H1N1) 2009, Mexico	Lack of information on system features
	Leal et al. (2020)	Prioritizing COVID-19 tests based in Participatory Surveillance and Spatial Scanning.	Wrong study design
PMID: 33002805	Liu et al. (2020)	Elucidating user behaviours in a digital health surveillance system to correct prevalence estimates	Lack of information on system features
N/A	Marinsek et al. (2020)	Measuring COVID-19 and Influenza in the Real World via Person-Generated Health Data	Non-participatory influenza surveillance system
PMID: 373163010	McIver & Brownstein, 2014	Wikipedia Usage Estimates Prevalence of Influenza-Like Illness in the United States in Near Real-Time	Non-participatory surveillance systems
PMID: 32410384	Monamele et al. 2020	Evaluation of a mobile health approach to improve the Early Warning System of influenza surveillance in Cameroon	Non-participatory surveillance systems
PMID: 24274205	Mulpuru et al. 2013	Evaluation of 3 electronic methods used to detect influenza diagnoses during 2009 pandemic	Non-participatory surveillance systems
PMID: 21894257	Olson et al. 2011	Applying a New Model for Sharing Population Health Data to National Syndromic Influenza Surveillance: DiSTRIBuTE Project Proof of Concept, 2006 to 2009	Non-participatory surveillance systems
PMID: 23462032	Paterson et al. 2013	Pandemic response in low-resource settings requires effective syndromic surveillance	Non-participatory surveillance systems
PMID: 29026453	Paul et al. 2017	Modeling and Forecasting Influenza-like Illness (ILI) in Houston, Texas Using Three Surveillance Data Capture Mechanisms	Non-participatory surveillance systems
PMID: 31169347	Rath et al. 2019	Partnering for enhanced digital surveillance of influenza-like disease and the effect of antivirals and vaccines (PEDSIDEA)	Non-participatory surveillance systems

PMID: 21672242	Reeder et al. 2011	Perceived usefulness of a distributed community-based syndromic surveillance system: a pilot qualitative evaluation study	Non-participatory surveillance systems
PMID: 20173026355	Robertson & Yee 2016	Avian influenza risk surveillance in North America with online media	Lack of information on system features
PMID: 26513245	Santillana 2015	Combining Search, Social Media, and Traditional Data Sources to Improve Influenza Surveillance.	Wrong study design
PMID: 18999051	South et al. 2008	Optimizing A syndromic surveillance text classifier for influenza-like illness: Does document source matter?	Non-participatory surveillance systems
PMID: 29208062	Souty et al. 2018	Performances of statistical methods for the detection of seasonal influenza epidemics using a consensus-based gold standard	Non-participatory surveillance systems
PMID: 15103900	Suzuki et al. 2004	[Evaluation of public knowledge about influenza based on influenza hotline consultations]	Non-participatory surveillance systems
PMID: 34623760	Temte et al. 2022	The Oregon Child Absenteeism Due to Respiratory Disease Study (orchards): rationale, objectives, and design.	Non-participatory influenza surveillance system
PMID: 24776527	Timpka et a. (2014)	Performance of eHealth data sources in local influenza surveillance: a 5-year open cohort study	Lack of information on system features
PMID: 24700999	Vong et al. 2014	Early response to the emergence of influenza A(H7N9) virus in humans in China: the central role of prompt information sharing and public communication	Wrong study design
PMID: 27372953	Wang et al. 2016	Regional Level Influenza Study with Geo-Tagged Twitter Data	Non-participatory surveillance systems
PMID: 20173273213	Xin et al. 2017	Analysis of influenza surveillance in Xinjiang, 2010-2015	Full text unavailable
PMID: 31170955	Yazidi et al. 2019	Evaluation of the influenza-like illness surveillance system in Tunisia, 2012-2015	Non-participatory surveillance systems

Table S1: Study Characteristics of 39 articles included in the review

Author (year)	Title	Location	Study Design	Study Period	Study Aim	Definition of ILI
Ackley *et al.* (2020)	Assessing the utility of a smart thermometer and mobile application as a surveillance tool for influenza and influenza-like illness	California, USA	Observational case study	2015–2016, 2016–2017	To evaluate the correlation between the regional California surveillance data and smart thermometer data.<br>To test the hypothesis that smart thermometer readings and symptom reports provide regionally specific predictions.<br>To determine whether smart thermometer and mobile application improved disease forecasts	Any illness with fever (greater than 37.8°C for oral readings) and cough and/or sore throat in the absence of a known cause other than influenza
Baltrusaitis *et al*. (2017)	Determinants of Participants' Follow-Up and Characterization of Representativeness in Flu Near You (FNY), A Participatory Disease	USA	Observational case study	2012-2015	To evaluate the representativeness of the FNY population compared with the general population of the United States.<br>To explore the demographic and behavioral characteristics associated with FNY's	Reported fever, cough, sore throat, shortness of breath, chills or night sweats, fatigue, nausea or vomiting, diarrhea, headache, and body ache

	Surveillance System				high-participation users. To summarize results from a user survey of a cohort of FNY participants	
Baltrusaitis *et al*. (2019)	Differences in Regional Patterns of Influenza Activity Across Surveillance Systems in the United States: Comparative Evaluation	USA	Observational case study	2015-2019	To calculate ILI proportions on regional and national level	Fever (temperature of 100°F [37.8°C] or greater) plus a cough and/or a sore throat without a known cause other than influenza
Bexelius *et al.* (2010)	Interactive Voice Response (IVR) and web-based questionnaires for population-based infectious disease reporting	Sweden	Cohort Study	2007-2008	To evaluate internet use and an IVR phone service as vehicles for surveillance	N/A
Biggerstaff *et al.* (2012)	Self-reported influenza-like illness and receipt of influenza antiviral drugs during the 2009 pandemic, United States, 2009-2010	USA	Observational case study	Sep 2009- Mar 2010	To describe the design and implementation of a community survey administered via the Behavioral Risk Factor Surveillance System (BRFSS) and present surveillance data collected from September 2009 to March 2010 in the United States	Fever with cough or sore throat in the 30 days preceding the BRFSS interview

Carlson *et al.* (2013)	Flu tracking weekly online community survey of influenza-like illness annual report 2011 and 2012	Australia	Cross-sectional (Survey)	2011-2012	To report on the 2011 and 2012 findings from the Flutracking ILI surveillance system	Fever and cough in preceding week
Cawley *et al.* (2021)	Novel Methods in the Surveillance of Influenza-Like Illness in Germany Using Data From a Symptom Assessment App (Ada): Observational Case Study	Germany	Observational case study	2017-2020	To explore the potential contribution of Ada data to syndromic surveillance, by comparing symptoms of ILI entered by Ada users in Germany with data from a national population-based reporting system called GrippeWeb	Fever with either cough or sore throat
Chunara *et al.* (2015)	Estimating influenza attack rates in the United States using a participatory cohort	USA	Observational case study	2012-2014	To investigate how participatory data can be used to estimate disease burden, specifically using Flu Near You surveillance data to estimate U.S. influenza attack rates	Presence of fever and either cough or sore throat
Cooper *et al.* (2008)	Can syndromic thresholds provide early warning of national influenza outbreaks?	England and Wales, UK	Observational case study	2002-2007	To better understand the value of syndromic data and test whether NHS Direct threshold levels for influenza surveillance provide advanced warning of community rises in	N/A

					influenza.	
Dalton *et al.* (2009)	Flutracking: A weekly Australian community online survey of influenza-like illness in 2006, 2007 and 2008	Australia	Cross – sectional (Survey)	2006-2008	To pilot an online community survey and assess its acceptability and feasibility for detecting inter-pandemic and, potentially, pandemic influenza in a regional health service with a population of 800,000 in south-eastern Australia during 2006 and expanded the project nationally in 2007 and 2008	Experience of cough, fever
Dalton *et al.* (2011)	Flutracking weekly online community survey of influenza-like illness annual report, 2010	Australia	Cross-sectional (Survey)	2010	To report on the 2010 findings from the Flutracking ILI surveillance system, including participation numbers compared with previous years, participant vaccination uptake for both the H1N1 pandemic (H1N1) 2009 monovalent and seasonal trivalent influenza vaccines, field vaccine effectiveness (FVE) estimates, weekly estimates of ILI and comparison of these estimates with other	N/A

					Australian influenza surveillance systems	
Dalton *et al.* (2015)	Flutracking weekly online community survey of influenza-like illness 2013-2014	Australia	Cross-sectional (Survey)	2013-2014	To report on the 2013-2014 findings from Flutracking regarding participation in the system, ILI incidence and vaccination rates	Fever and cough
Debin *et al.* (2013)	Evaluating the feasibility and participants' representativeness of an online nationwide surveillance system for influenza in France	France	Observational case study	Jan 25th - April 29th, 2012	To establish feasibilty of an online participatory surveillance system by evaluating recruitment, participation and representativeness in GrippeNet.fr (GN) under consideration of the representativeness of the study population	N/A
de Lange *et al*. (2013)	Comparison of five influenza surveillance systems during the 2009 pandemic and their association with media attention	The Netherlands	Observational case study	April 20th, 2009- January 3rd, 2012	To compare ILI incidence among five surveillance systems and to compare ILI dynamics with media coverage regarding influenza	Sudden onset of fever accompanied by muscle pain and cough and/or sore throat and/or chest pain
Desroches *et al*. (2021)	Representativeness of the FluWatchers Participatory Disease Surveillance Program 2015-	Canada	Cross-sectional	2015-2019	To evaluate the representativeness of the most recent FluWatchers participant population against the Canadian population.	N/A

	2016 to 2018-2019: How do participants compare with the Canadian population?				To understand changes in representativeness since its pilot in 2015.<br>To characterize a typical FluWatcher to identify gaps and biases.	
Doroshenko *et al.* (2005)	Evaluation of syndromic surveillance based on National Health Service Direct derived data--England and Wales	England, Wales	Observational case study	2001-2004	To evaluate NHS Direct syndromic surveillance using the "Framework for Evaluating Public Health Surveillance Systems for Early Detection of Outbreaks", published by CDC.	N/A
Elliot *et al.* (2015)	Self-sampling for community respiratory illness: a new tool for national virological surveillance	UK	Cross-sectional	May 2009-Mar 2010	To evaluate the usefulness of self-sampling for surveillance of emerging respiratory infections	N/A
Fujibayashi *et al.* (2018)	A New Influenza-Tracking Smartphone App (Flu-Report) Based on a Self-Administered Questionnaire: Cross-Sectional Study	Japan	Cross-sectional	November 2016-March 2017	To evaluate the flu-tracking ability of Flu-Report, a new influenza-tracking mobile phone app that uses a self-administered questionnaire for the early detection of influenza activity	N/A

Guerrisi *et al.* (2018)	The potential value of crowdsourced surveillance systems in supplementing sentinel influenza networks: the case of France	France	Cross-sectional (Survey)	2012-2014	To explore the usefulness of data from GN, a participatory surveillance system, to estimate influenza-related illness incidence in France	Sudden onset of fever (>39) AND pain with sore throat OR cough OR shortness of breath OR sneezing OR rhinorrhea
Kamimoto *et al*. (2013)	Seasonal influenza morbidity estimates obtained from telephone surveys, 2007	USA	Observational case study	2006-2007	To assess telephone surveys as a novel surveillance method, comparing data obtained by telephone with existing national influenza surveillance systems.<br>To evaluate the utility of telephone surveys.	Self-reported fever in addition to cough, sore throat, or both
Kim *et al.* (2019)	The Fever Coach Mobile App for Participatory Influenza Surveillance in Children: Usability Study	South Korea	Cross-sectional	2016-2018	To evaluate the use of parent-reported data of febrile illnesses in children submitted through the Fever Coach app in real-time surveillance of influenza activities	Fever equal to or greater than 38.0°C (100.4°F) accompanied by cough or sore throat
Kjelso *et al.* (2015)	Influmeter – an online tool for self-reporting of influenza-like illness in	Denmark	Cross-sectional (Survey)	2013-2014	To evaluate the system use in terms of representativeness in the Danish population, compliance to report	Any symptom of fever or feverishness (chills) OR malaise, headache or muscle pain AND at least one of the following symptoms: cough, sore throat or shortness of breath

	Denmark				symptoms throughout the season and benefit to existing ILI surveillance in Denmark.	
Lee *et al.* (2014)	Respiratory symptoms in households as an effective marker for influenza-like illness surveillance in the community	Tuen Mun District, Hong Kong,China	Cross-sectional (Random sampling Survey)	2012-2013	To test the feasibility of community-level ILI surveillance and to evaluate the validity of selected syndromic markers for assessing temporal trends	Fever above 38◦C and cough or sore throat
Lee *et al.* (2021)	FluWatchers: Evaluation of a crowdsourced influenza-like illness surveillance application for Canadian influenza seasons 2015-2016 to 2018-2019	Canada	Observational case study	2015-2019	To evaluate FluWatchers, a crowdsourced ILI application developed to complement and complete ILI surveillance in Canada	A report of fever and cough.
Loubet *et al.* (2016)	First nationwide web-based surveillance system for influenza-like illness in pregnant women: participation and representativeness of the French G-	France	Observational case study	2014-2015	To assess the representativeness and participation of French women to a new web-based collaborative tool for data collection and monitoring of ILI during pregnancy	(i) The sudden onset of symptoms, (ii) at least one of the following signs: fever or chills or headache or myalgia or asthenia and (iii) at least one of the following respiratory symptoms: cough, sore throat, shortness of breath (dyspnea)

	GrippeNet (GGNET) cohort					
Lucero *et al.* (2017)	Enhanced Influenza Surveillance Using Telephone Triage (TT) and Electronic Syndromic Surveillance in the Department of Veterans Affairs, 2011-2015	USA	Cross-sectional (Survey)	2011-2015	To systematically evaluate the use and timeliness of TT	N/A
Lwin *et al.* (2020)	Effectiveness of a Mobile-Based Influenza-Like Illness Surveillance System (FluMob) Among Health Care Workers: Longitudinal Study	Singapore	Observational case study	2016-2018	To assess the efficiency of a mobile-based surveillance system of ILI, termed FluMob, among health care workers using a targeted surveillance approach.<br>To evaluate the effectiveness of the system for ILI surveillance pertaining to its participation engagement and surveillance power.<br>To identify the factors that can moderate the effectiveness of the system.	Fever (≥38.0°C) accompanied by a cough or a sore throat

Ma *et al.* (2015)	Syndromic surveillance of influenza activity in Sweden: an evaluation of three tools	Sweden	Observational case study	2009-2013	To determine which syndromic surveillance tools complement traditional surveillance by serving as earlier indicators of influenza activity in Sweden	Fever
Marmara *et al.* (2021)	Cross-sectional telephone surveys as a tool to study epidemiological factors and monitor seasonal influenza activity in Malta.	Malta	Cross-sectional (Survey)	2014-15, 2015-16	To study seasonal influenza dynamic in Malta in 2014-16, including social dynamics and self-perception of the seasonal flu	Sudden onset, fever>38°C and other symptoms
Miller *et al.* (2018)	A Smartphone-Driven Thermometer Application for Real-time Population- and Individual-Level Influenza Surveillance	California, USA	Observational case study	2015-2017	To explore the utility of using large-scale geocoded data from commercially available smart thermometers to perform population- and individual-level influenza surveillance	Temperature 100°F (37.8°C) or greater and a cough and/or a sore throat without a known cause other than influenza
Paixao *et al.* (2013)	Improving influenza surveillance in Portuguese preschool children by parents' report	Portugal	Observational case study	2012	To test a supplementary surveillance system based on parent reports	fever, wheezing, cough or nasal congestion
Perrotta *et al.* (2017)	Participatory Online	Italy	Observational case study	2012-2015	To explore how Influweb operated between 2012-15	Sudden onset + at least one from the following: fever, chills, tiredness,

	Surveillance as a Supplementary Tool to Sentinel Doctors for Influenza-Like Illness Surveillance in Italy.				compared to the standard surveillance methods	headache, muscle/joint ache + at least one from the following: sore throat, cough, shortness of breath
Prieto *et al.* (2017)	Will Participatory Syndromic Surveillance Work in Latin America? Piloting a Mobile Approach to Crowdsource Influenza-Like Illness Data in Guatemala	Guatemala	Coss-sectional (Cluster-random sampling Survey)	2016-2017	To evaluate the quality and characteristics of electronically collected data, the user acceptability of the symptom reporting platform, and the costs of running the system and of identifying ILI cases.<br>To use the collected data to characterize cases of reported ILI	Fever and cough or sore throat, with onset within the last 7 days.
Rehn *et al.* (2014)	Evaluation of an Internet-based monitoring system for influenza-like illness in Sweden	Sweden	Observational case study	Nov 2011-May 2012, Nov 2012-May 2013	To evaluate an internet-based monitoring system in comparison with a previously evaluated population-based system	Sudden onset of symptoms AND at least one of the following systemic symptoms: fever or feverishness, headache, or myalgia, AND at least one of the following respiratory symptoms: cough, sore throat, shortness of breath.
Stockwell *et al*. (2014)	MoSAIC: Mobile Surveillance for Acute Respiratory Infections and Influenza-Like Illness in the Community	NYC, USA	Cross-sectional (Random sampling Survey)	2012-2013	To assess the feasibility of using text messaging to conduct ILI/ARI surveillance in a US community and the impact of text messaging on timeliness of illness	N/A

					reporting and specimen collection	
Takahashi *et al.* (2001)	Evaluation of the Japanese School Health Surveillance Systems for Influenza	Japan	Observational case study	1998-1999	To determine the efficacy of the Japanese School Health Surveillance System for ILI outbreaks	N/A
Tilston *et al.* (2010)	Internet-based surveillance of Influenza-like-illness in the UK during the 2009 H1N1 influenza pandemic	Great Britain (England, Wales, Scotland and Northern Ireland)	Cross sectional	2009	To assess how well internet-based surveillance of Influenza performs when compared to a range of other measures.	Sudden onset of fever with muscle pain accompanied by either a cough, sore throat or chest pain
Vandendijck *et al.* (2013)	Eight years of the Great Influenza Survey (GIS) to monitor influenza-like illness in Flanders	Flander, Belgium	Cross-sectional (Survey)	2003-2009	To assess the validity of the GIS in Flanders with respect to the trend estimation of ILI incidence and the representativeness of the survey population.	Sudden onset of fever, namely, a measured body temperature of 38°C or more, accompanied with headache or muscle pain and accompanied with cough or a sore throat
van Noort *et al.* (2015)	Ten-year performance of Influenzanet: ILI time series, risks, vaccine effects, and care-seeking behaviour	Netherlands, Belgium, Portugal, and Italy	Observational case study	2003-2013	To further establish the Influenzanet system as a valid sentinel for ILI surveillance, by confirming that both the timing and relative intensities of epidemics are consistent with those reported by ECDC, and the identified risk factors for	Acute onset (within a few hours) of fever (a measured temperature of at least 38 °C), together with muscle pain or headache, and cough or sore throat.

					ILI are consistent with those in the published literature.	

Table S2: Quality assessment of 3 cohort studies included in the review

Author (year)	Were the two groups similar and recruited from the same population?	Were the exposures measured similarly to assign people to both exposed and unexposed groups?	Was the exposure measured in a valid and reliable way?	Were confounding factors identified?	Were strategies to deal with confounding factors stated?	Were the groups/participants free of the outcome at the start of the study (or at the moment of exposure)?	Were the outcomes measured in a valid and reliable way?	Was the follow up time reported and sufficient to be long enough for outcomes to occur?	Was follow up complete, and if not, were the reasons to loss to follow up described and explored?	Were strategies to address incomplete follow up utilized?	Was appropriate statistical analysis used?	Overall Study Quality
Bexelius *et al.* (2010)	Yes	N/A	N/A	Yes	Yes	Yes	No	Yes	Yes	Yes	Yes	High
Elliot *et al.* (2015)	Yes	N/A	Unclear	Yes	Yes	Yes	Yes	Yes	Yes	N/A	Yes	High
Paixao *et al.* (2014)	Yes	N/A	N/A	Yes	Unclear	Yes	Yes	Yes	Yes	Unclear	Yes	High

Table S3: Quality assessment of 36 cross-sectional/ observational case studies included in the review

Author (year)	Was the sample frame appropriate to address the target population?	Were study participants sampled in an appropriate way?	Was the sample size adequate?	Were the study subjects and the setting described in detail?	Was the data analysis conducted with sufficient coverage of the identified sample?	Were valid methods used for the identification of the condition?	Was the condition measured in a standard, reliable way for all participants?	Was there appropriate statistical analysis?	Was the response rate adequate, and if not, was the low response rate managed appropriately?	Overall Study Quality
Ackley *et al.* (2020)	Yes	No	Yes	No	Yes	Yes	No	Yes	Yes	Moderate
Baltrusaitis *et al*. (2017)	Yes	Yes	Yes	Yes	Yes	No	No	Yes	Yes	Moderate
Baltrusaitis *et al*. (2019)	Yes	Yes	Yes	Yes	Yes	No	No	Yes	Yes	Moderate
Biggerstaff *et al*. (2012)	Yes	Yes	Yes	Yes	Yes	No	No	Yes	Yes	Moderate
Carlson *et al.* (2013)	Yes	Yes	Yes	Yes	Yes	No	No	Yes	Yes	Moderate
Cawley *et al.* (2021)	Yes	Yes	Yes	Yes	Yes	No	No	Yes	Yes	Moderate
Chunara *et al.* (2015)	Yes	Yes	Yes	Yes	Yes	No	No	Yes	Yes	Moderate
Cooper *et al.* (2008)	Yes	Yes	Unclear	No	Yes	No	No	Yes	Yes	Moderate
Dalton *et al.* (2009)	Yes	Yes	Yes	Yes	Yes	No	No	Yes	Yes	Moderate

Dalton *et al.* (2011)	Yes	Yes	Yes	Yes	Yes	No	No	Yes	Yes	Moderate
Dalton *et al.* (2015)	Yes	Yes	Yes	Yes	Yes	No	No	Yes	Yes	Moderate
Debin *et al.* (2013)	Yes	Yes	Yes	Yes	Yes	No	No	Yes	Yes	Moderate
de Lange *et al.* (2013)	Yes	Yes	Unclear	Yes	Unclear	Yes	No	Yes	Unclear	Moderate
Desroches *et al.* (2021)	Yes	No	No	Yes	No	No	Yes	Yes	Yes	Moderate
Doroshenko *et al.* (2005)	Yes	No	No	Yes	Yes	Yes	No	Yes	Yes	Moderate
Fujubayashi *et al.* (2018)	Yes	Yes	Yes	Yes	No	No	No	Unclear	Yes	Moderate
Guerrisi *et al.* (2018)	Yes	Yes	Yes	Yes	Yes	No	No	Yes	Yes	Moderate
Kamimoto *et al.* (2013)	Yes	Yes	No	Yes	Yes	Yes	No	Yes	Yes	Moderate
Kim *et al.* (2019)	Yes	Yes	Yes	Yes	Yes	No	No	Yes	Yes	Moderate
Kjelso *et al.* (2015)	Yes	Yes	Yes	Yes	Yes	No	No	Yes	Yes	Moderate
Lee *et al.* (2014)	Yes	Yes	Yes	Yes	Yes	No	No	Yes	Yes	Moderate
Lee *et al.* (2021)	Yes	Yes	Unclear	No	Yes	No	No	Yes	Yes	Moderate
Loubet *et al.* (2016)	No	No	No	Yes	Yes	Yes	Yes	Yes	No	Moderate

Lucero-Obusan *et al.* (2017)	No	No	Yes	Yes	Yes	No	Yes	Yes	Yes	Moderate
Lwin *et al.* (2020)	Yes	Yes	No	Yes	Yes	Yes	No	Yes	Yes	Moderate
Ma *et al.* (2015)	Yes	Yes	Yes	Yes	Yes	No	No	Yes	Yes	Moderate
Marmara *et al.* (2021)	Yes	Yes	Yes	Yes	Yes	Yes	Unclear	No	Yes	Moderate
Miller *et al.* (2018)	Yes	Yes	Yes	Yes	Yes	No	No	Yes	Yes	Moderate
Perrotta *et al.* (2017)	Yes	Yes	Unclear	Yes	Unclear	Yes	No	Yes	Unclear	Moderate
Prieto *et al.* (2017)	Yes	Yes	Yes	Yes	Yes	No	No	Yes	Yes	Moderate
Rehn *et al.* (2014)	Yes	Yes	Unclear	Yes	Yes	No	No	Yes	Yes	Moderate
Stockwell *et al.* (2014)	Yes	Yes	Yes	Yes	Yes	No	No	Yes	Yes	Moderate
Takahashi *et al.* (2001)	Yes	Yes	Yes	Unclear	Yes	Unclear	Unclear	Yes	Yes	Moderate
Tilston *et al.* (2010)	Yes	Yes	Yes	Yes	Yes	No	No	Yes	Yes	Moderate
Vandendijck *et al.* (2013)	Yes	Yes	Yes	Yes	Yes	No	No	Yes	Yes	Moderate
van Noort *et al.* (2015)	Yes	Yes	Yes	No	Yes	No	No	Yes	Yes	Moderate

Table S4: Attributes of each system discussed in the studies included

Author (year)	Location	Name of System	Attributes of System						
			Acceptability	Completeness	Correlation/ Concurrence with other surveillance systems	Flexibility	Representativeness	Sensitivity	Simplicit
Ackley *et al.* (2020)	California, USA	Kinsa Smart thermometer and mobile application			•				
Baltrusaitis *et al.* (2017), Baltrusaitis *et al.* (2019), Chunara *et al.* (2015),	USA	Flu Near You			•				
Bexelius *et al.* (2010)	Sweden	No actual name, "Interactive Voice Response and web-based questionnaires"				•			
Biggerstaff *et al.* (2012)	USA	BRFSS	•						

Carlson *et al*. 2013, Dalton *et al*. (2009), Dalton *et al*. (2011), Dalton *et al*. (2015)	Australia	Flu tracking	•		•		•		
Cawley *et al*. (2021)	Germany	Ada			•		•		
Cooper *et al*. (2008)	England and Wales, UK	National Health Service (NHS) Direct			•			•	
Debin *et al*. (2013)	France	GN	•		•				
Desroches *et al*. (2021), Lee *et al*. (2021)	Canada	Fluwatchers	•		•		•		
Doroshenko *et al*. (2005)	England/Wales	NHS Direct syndromic surveillance		•			•	•	
Elliot *et al*. (2015)	UK	National Pandemic Flu Service							
Fujibayashi *et al*.	Japan	Flu-Report			•		•		

(2018)									
Guerrisi *et al.* (2018)	France	GrippeNet.fr			•		•		
Kamimoto *et al.* (2013)	USA	BRFSS, National Immunization Survey–Adult (NIS–Adult)			•				
Kim *et al.* (2019)	South Korea	Fever Coach			•			•	
Kjelso *et al.* (2015)	Denmark	Influmeter	•				•	•	
Lee *et al.* (2014)	Tuen Mun District, Hong Kong	E-community Surveillance System for Influenza-Like Illness					•	•	
Loubet *et al.* (2016)	France	G-GNET				•			
Lucero *et al.* (2017)	USA	US Department of Veterans Affairs, Telephone Triage (VA TT) ESSENCE			•	•			
Lwin *et al.* (2020)	Singapore	FluMob			•				

Ma *et al.* (2015)	Sweden	Medical telephone hotline	•		•				
Marmara *et al.* (2015)	Malta	Survey 1 and Survey 2			•				
Miller *et al.* (2018)	California, USA	Kinsa Smart Thermometer			•			•	
Paixao *et al.* (2017)	Portugal	No actual name, “Surveillance project based/ nested within Environment and Health in children day care centers (ENVIRH)”							
Perrota *et al.* (2017)	Italy	Influweb							
Prieto *et al.* (2017)	Guatemala	Mi Gripe	•	•	•	•			
Rehn *et al.* (2014)	Sweden	IMS (Swedish Version of Influenzanet)							
Stockwell *et al.* (2014)	NYC, USA	MoSAIC							
Takahashi *et al.* (2001)	Japan	Japanese School Health Surveillance System for Influenza					•		

Tilston *et al.* (2010)	Great Britain (England, Wales, Scotland and Northern Ireland)	UK Flu Survey			•				
Vandensjick *et al.* (2013), de Lange *et al.* (2013)	Belgium	GIS	•		•		•		
van Noort *et al.* (2015)	the Netherlands, Belgium, Portugal, and Italy	Influenzanet			•	•			
Total			8	2	20	6	10	6	

Table S5: ILI Surveillance Systems' Approaches to Collecting Data

Author (year)	Location	System	Data Collection	Data Storage
Ackley *et al.* (2020)	California, USA	Kinsa Smart thermometer and mobile application	Data collected from the smart thermometer and mobile application	N/A
Baltrusaitis e*t al.* (2017), Baltrusaitis *et al.* (2019), Chunara *et al.* (2015)	USA	FNY	Data on self-reported symptoms among participants in the Flu Near You (FNY) surveillance platform (flunearyou.org) in the United States during the 2012–2013 and 2013–2014 influenza seasons were used	N/A
Bexelius *et al.* (2010)	Sweden	IVR and web-based questionnaires	Data collected via a study-specific website or interactive voice response system	N/A
Biggerstaff *et al.* (2012)	USA	BRFSS	Data collected via telephone interview of randomly selected participants	N/A
Carlson *et al.* 2013, Dalton *et al.* (2009), Dalton *et al.*(2011)	Australia	Flu tracking	Data collected from participants who completed the survey	N/A
Cawley *et al.* (2021)	Germany	Ada	Data from all Ada assessments (i.e. for any symptoms or complaints) completed by users	N/A

			in Germany between calendar week 27 2017 and calendar week 26 2020 were extracted	
Cooper *et al.* (2008), Doroshenko *et al.* (2005)	England and Wales, UK	NHS Direct	Data collected from calls to the NHS Direct.	N/A
Dalton *et al.* (2015)	Australia	Flutracking	Data collected via weekly surveys distributed among the recruited cohort	N/A
Debin *et al.* (2013); Guerrisi *et al.* (2018)	France	GN	Data were collected through website https://www.grippenet.fr	N/A
de Lange *et al.* (2013)	The Netherlands	GIS	Data collected via weekly e-mail prompts with links to survey asking whether 11 prespecified symptoms occurred since the last visit to the website - if yes, then additional questions.	N/A
Desroches *et al.* (2021), Lee *et al.* (2021)	Canada	Fluwatchers	Data collected from a brief, weekly symptom-based report via an anonymous online questionnaire	N/A
Espino *et al.* (2003)	Pennsylvania, USA	Telephone Triage	Secondary data analysis of two datasets from a healthcare call center services and software company	N/A
Elliot *et al.* (2015)	UK	National Pandemic Flu Service	Data collected web-based and by telephone plus self-administered samples	N/A

Fujibayashi *et al.* (2018)	Japan	Flu-Report	Data collected using a self-administered questionnaire	N/A
Kamimoto *et al.* 2013	USA	Telephone surveys	Data collected from BRFSS and NIS-Adult during 2006-2007 influenza season	N/A
Kim *et al.* (2019)	South Korea	Fever Coach	Data submitted to the app during 2016-2017, and 2017-2018 influenza seasons were used.	All user-reported data were stored in Fever Coach databases in real time, and metadata (number of submissions and location information) on each day were processed and saved daily
Kjelso *et al.* (2015)	Denmark	Influmeter	Data were collected from the web-based self-reporting system, influmeter	N/A
Lee *et al.* (2014)	Tuen Mun District, Hong Kong	E-community Surveillance System for Influenza-Like Illness	Data provided by participants on any reported fever and/or respiratory symptoms (cough, runny nose, sore throat) in the preceding week, both for themselves and for their household, were used	N/A
Loubet *et al.* *(*2016)	France	GGNET	Participants register through their email on the Grippe net website and submit weekly self-reported questionnaires	
Lucero *et*	USA	VA-	Web based standardized decision-	N/A

al.(2017)		TT ESSENCE	making triage system for TT nurses. Used structured protocol and algorithms to assess the patient condition	
Lwin *et al.* (2020)	Singapore	FluMob	Data collected using an online questionnaire for ILI symptoms sent through app notifications	Data is stored in real time in the central database and can be accessed by administrators via an analytical module integrated into the central servers
Ma *et al.* (2015)	Sweden	Hotline	Web queries algorithm: The algorithm, based on 20 types of influenza-related key terms (e.g. fever) with their corresponding weights, is computed every week to capture influenza activity in a manner that mimics the case definition reported by the sentinel reporting tool.	N/A
Marmara *et al.* (2015)	Malta	Survey 1 and Survey 2	Information was collected using a questionnaire	N/A
Miller *et al.* (2018)	California, USA	Kinsa Smart Thermometer	Kinsa Smart Thermometer was used to capture ILI activity using three thermometer readings: (1) weekly counts of total temperature readings; (2) total fever readings (ie, temperature ≥37.8°C	N/A

			and (3) the total number of distinct fever episodes, identified by distinct user profiles registering a fever in each week.	
Perrotta *et al*. (2017)	Italy	Influweb	Participants log on the platform and get anonymised, password-protected accounts, weekly syndromic surveys completed by the participants (weekly reminders sent)	N/A
Paixao *et al.* (2014)	Portugal	Surveillance project based/ nested within Environment and Health in children day care centers (ENVIRH)"	Data collected by phone from parents plus samples taken by dedicated study team	N/A
Prieto *et al.* (2017)	Guatemala	Mi Gripe	The researchers created a bimodal data capturing system which was capable of capturing ILI reports from SMS and Mi Gripe	N/A
Rehn *et al.* (2014)	Sweden	IMS (Swedish Version of Influenzanet)	Data is collected online following weekly email-reminders	N/A
Stockwell *et al.* (2014)	NYC, USA	MoSAIC	Data were collected from household report text messages	N/A
Takahashi *et al.* (2001)	Japan	Japanese School Health Surveillance	Data taken from School Health Surveillance System (SHSS)	N/A

		System for Influenza		
Tilston *et al.* (2010)	Great Britain (England, Wales, Scotland and Northern Ireland)	UK Flu Survey	Data were collected via the symptom's questionnaire	N/A
Vandensjick *et al.* (2013)	Belgium	GIS	Data collected from online questionnaire	N/A
van Noort *et al.* (2015)	the Netherlands, Belgium, Portugal, and Italy	Influenzanet	Data collected from an online weekly questionnaire sent to patients during the influenza season.	N/A

Table S6: ILI Surveillance Systems' Study Approval

Author (year)	Location	System	Study Approval by Ethics Board	Study Approval by Informed Consent (Yes/No/ NA)
Ackley *et al.* (2020)	California, USA	Kinsa Smart thermometer and mobile application	Institutional Review Boards at University of California, San Francisco and the California Department of Public Health	N/A
Bexelius *et al*. (2017)	Sweden	No actual name, "Interactive Voice Response and web-based questionnaires"	N/A	Yes

Cawley *et al.* (2021)	Germany	Ada	N/A	Yes
Cooper *et al.* (2008), Doroshenko *et al.* (2005)	England and Wales, UK	NHS Direct	N/A	N/A
Debin *et al.* (2013), Guerrisi *et al.* (2018), Loubet *et al.* (2016)	France	GN	French Advisory Committee for research on information treatment in the field of health and by the French National Commission on Informatics and Liberty	N/A
de Lange *et al.* (2013)	The Netherlands	GIS	N/A	N/A
Desroches *et al.* (2021), Lee *et al.* (2021)	Canada	Fluwatchers	N/A	N/A
Elliot *et al.* (2015)	UK	National Pandemic Flu Service	N/A	Yes
Fujibayashi *et al.* (2018)	Japan	Flu-Report	Ethical Review Board of Juntendo University	Yes
Kim *et al.* (2019)	South Korea	Fever Coach	Institutional Review Board of CHA University	Yes
Lee *et al.* (2014)	Tuen Mun District, Hong Kong	E-community Surveillance System for Influenza-Like Illness	Chinese University of Hong Kong Committee on Survey and Behavioural Research Ethics	Yes

Loubet *et al.* (2016)	France	G-GNET	Comité consultatif sur le traitement de l'information en matière de recherche (CCTIRS, Advisory committee on information processing for research, authorisation) and by the Commission Nationale de l'Informatique et des Libertés (CNIL, French Data Protection Authority).	Yes
Lwin *et al.* (2020)	Singapore	Flu Mob	National Health Group Domain Specific Review Board and the SingHealth Centralised Institutional Review Board	Yes
Marmara *et al.* (2015)	Malta	Survey 1 and Survey 2	Psychology Ethics Committee at University of Stirling	Yes
Miller *et al.* (2018)	California, USA	Kinsa Smart Thermometer	University of Iowa Institutional Review Board	No
Paixao *et al.* (2014)	Portugal	No actual name, "Surveillance project based/ nested within Environment and Health in children day care centers (ENVIRH)"	Ethics Committee of Nova Medical School, Lisbon	

Perrotta *et al.* (2017)	Italy	Influweb	N/A	N/A
Prieto *et al.* (2017)	Guatemala	Mi Gripe	Ethics Committee of Universidad del Valle de Guatemala on July 5, 2016, and by the Guatemalan National Ethics Committee on August 5, 2016.	Yes
Rehn *et al.* (2014)	Sweden	IMS (Swedish Version of Influenzanet)	Stockholm regional research ethics review board (IMS: 2011/387-31/4, 2012/1445-32/4)	Yes
Stockwell *et al.* (2014)	NYC, USA	MoSAIC	Institutional review boards of the Columbia University Medical Center and the Centers for Disease Control and Prevention (Atlanta, Georgia)	N/A
Tilston *et al.* (2010)	Great Britain (England, Wales, Scotland and Northern Ireland)	UK Flu Survey	London School of Hygiene and Tropical Medicine Ethics Committee	N/A
Vandensjick *et al.* (2013)	Belgium	GIS	N/A	No
van Noort *et al.* (2015)	the Netherlands, Belgium, Portugal, and Italy	Influenzanet	Ethics Committee of Instituto Gulbenkian de Ciência	N/A

Table S7: ILI Surveillance Systems Methods for addressing bias and confounders

Author (year)	Location	System	Adjustment for potential bias	Adjustment for confounders
Ackley *et al.* (2020)	California, USA	Kinsa Smart thermometer and mobile application	N/A	In order to exclude temperature readings for causes other than ILI, users who measured temp > 500 times a year were excluded from the analysis
Baltrusaitis *et al.* (2017), Baltrusaitis *et al.* (2019), Chunara *et al.* (2015)	USA	FNY	To avoid double-counting of a single ILI episode researchers have discarded weekly ILI reports for individuals who have also reported ILI during the preceding week	N/A
Bexelius *et al.* (2017)	Sweden	No actual name, "Interactive Voice Response and web-based questionnaires"	N/A	Adjustments were made for gender
Cawley *et al.* (2021)	South Korea	Fever Coach	To filter out falsely submitted data, the researchers only used body weight values from 5 percentile of newborns (2.5 kg) to 95 percentile of 18-year-olds (79.8 kg)	N/A

			based on the Korean child growth curve in 2017.<br>As the age of a child changed over time, researchers calculated the mean of the age during each period if data were submitted multiple times.<br>For body weight, the researchers only considered the last submitted data for each child.	
Dalton *et al*. (2015)	Australia	Flu tracking	Survey designed to be free from responder bias caused by health seeking behaviour, from observer bias caused by differentiated GP testing practices or differentiated surveillance practices in various parts of the country	N/A
Debin *et al.* (2013), Guerrisi *et al.* (2018)	France	GN	The researchers performed first survey exclusion, which adjusts GN incidence rates by removing the result of the first survey of newly enrolled participants as participants are more prone to report symptoms at their first report following enrolment.<br>The researchers implemented a minimum number "m" of symptoms reports per individual throughout the season (m = 2 reports or m = 3 reports, including the first survey) to discard those with rare participation.<br>The researchers considered the inclusion criterion of a participation window of" n" weeks around the	Adjustments for age and regional geographic distributions were made

			reporting week (n = 0, 1, 2, 3, 4) to account for non-continuous participation. Incidence time series computed on these datasets were adjusted by age group to account for the non-representative nature of the GN population, and smoothed through a linear filtering method to filter out undesired spikes induced by large variations in enrolment.<br>Debin: short-term participation bias controlled for by only including participants who completed the intake questionnaire plus at least 3 surveys	
Kamimoto *et al.* (2013)	USA	BRFSS and NIS-Adult telephone surveys	Using a modified influenza questionnaire over two time periods for BRFSS, the researchers were able to assess the impact of one month over 3 to 11 months recall for self-reported influenza morbidity measures	N/A
Kim *et al.* (2019)	South Korea	Fever Coach	To filter out falsely submitted data, the researchers only used body weight values from 5 percentile of newborns (2.5 kg) to 95 percentile of 18 year olds (79.8 kg) based on the Korean child growth curve in 2017. The reseachers calculated the mean of the age during each period if data were submitted multiple times. For body weight, they only	N/A

			considered the last submitted data for each child.	
Kjelso *et al.* (2015)	Denmark	Influmeter	To avoid first-time reporting bias the researchers calculated Influmeter ILI rates discarding every participant’s first report.	N/A
Marmara *et al*. (2015)	Malta	Survey 1 and Survey 2	Second survey was carried out between weeks 17–19 as compared to weeks 35–37 in Survey 1 may have resulted in lower recall bias as respondents might have found it easier to recall their ILI symptoms	Both samples were stratified based on sex, district and age distribution for general population.
Perrotta *et al*. (2017)	Italy	Influweb	Only restricted sample analysed to reduce recruitment bias towards sick people. In order to avoid having a variable and biased sample, mainly due to the possibility for volunteers to join the platform throughout the influenza season, the researchers have definedd a subsample of users called active participants.	N/A
Rehn *et al.* (2014)	Sweden	IMS (Swedish Version of Influenzanet)	Weighting to account for selection bias	N/A
Tilston *et al.* (2010)	Great Britain (England, Wales, Scotland and Northern Ireland)	UK Flu Survey	Because of the demographic biases contained within sample, to allow comparison between influenza statistics derived using the Flusurvey data with those estimated for the UK, the researchers	N/A

			reweighed the sample in their analysis. To do this they weighed their sample according to age and gender. They split their samples into age categories < 1, 1-4, 5-14, 15-24, 25-44, 45-64 and 65+ to match the UK census age distribution.<br><br>To reduce the effect of individuals who only register and take part as a one-off response to their current symptoms, the researchers restricted their dataset to participants' second and subsequent reports.	
Vandensjick *et al.* (2013)	Belgium	Great Influenza Survey (GIS)	To reduce the effect of volunteers that only participated rarely and those who took part as a one-off response to their current symptoms, data from the first symptom questionnaire are excluded and only data of participants that completed at least three symptom questionnaires are used.<br>When a participant experienced ILI in two or more symptom questionnaires that are not separated by more than a fortnight, these symptoms are considered to belong to the same ILI episode.	N/A
Van Noort *et al.* (2015)	the Netherlands, Belgium, Portugal, and	Influenzanet	To minimize the selection bias in recruiting participants who already have ILI, any symptoms that started	N/A

	Italy		before or on the registration date were excluded from the analysis. Only participants who participated at least 3 times during a season were included in the analyses.	
